# *Parthenocissus*-inspired soft climbing robots

**DOI:** 10.1126/sciadv.adt9284

**Published:** 2025-03-26

**Authors:** Kecheng Qin, Wei Tang, Huaizhi Zong, Xinyu Guo, Huxiu Xu, Yiding Zhong, Yonghao Wang, Qincheng Sheng, Huayong Yang, Jun Zou

**Affiliations:** State Key Laboratory of Fluid Power and Mechatronic Systems, School of Mechanical Engineering, Zhejiang University, Hangzhou 310058, China.

## Abstract

Climbing robots have attracted growing attention due to their mobility on vertical and nonplanar structural surfaces. However, the development of climbing robots capable of climbing on various complex surfaces remains elusive, especially on discontinuous surfaces. In nature, *Parthenocissus* climbs as it grows, having growing-climbing behaviors. Inspired by *Parthenocissus*, we propose a growing-climbing mechanism and report a soft climbing robot, which grows microstructured biofilms to enhance adhesion, similar to *Parthenocissus* growing suckers and adsorbing to the wall. The robot uses shape memory alloy contraction to achieve bending, similar to *Parthenocissus* using gelatinous fibers contraction to achieve hinge-like bending. In addition, to not damage the site, it can be fully contracted after completing tasks. The climbing robot can climb on various complex surfaces, especially discontinuous surfaces, verifying the effectiveness of *Parthenocissus*’ growing-climbing mechanism. The growing-climbing mechanism is a universal climbing robot paradigm, opening a door for complex surface climbing robots.

## Teaser

*Parthenocissus*-inspired soft climbing robots, having growing-climbing behaviors, can climb on complex surfaces.

## INTRODUCTION

Climbing robots (*1*–*5*) have received widespread attention and have been widely used because of their ability to move on vertical surfaces, ceilings, or pipes. Thanks to their excellent antigravity movement capabilities, climbing robots are well-known for their movement performance on artificial and natural terrains. Some important scenarios, such as aviation maintenance, industrial manufacturing, nuclear power plant maintenance, and building cleaning, require climbing robots to have the ability to climb complex surfaces or even discontinuous surfaces. Here, complex surfaces are defined as surfaces that have both complex structures (curves, gaps, roughness, etc.) and complex materials (cement, metal, wood, etc.).

The two keys to climbing robots are the adhesion mechanism and the locomotion mechanism. Existing climbing robots use animal-inspired locomotion mechanisms (*1*–*3*, *6*–*9*), including legs, translation, inchworm motion, wheels, etc. Adhesion mechanisms (*10*–*18*) include vacuum suckers, magnets, electrostatic adsorption, mechanical clamping, dry adhesives, etc. The combination of different locomotion mechanisms and adhesion mechanisms constitutes the existing climbing robots. For example, quadruped robots can climb agilely in ferromagnetic environments using magnetic fluid adsorption, the combination of inchworm motion and external magnetic fields enables bionic crawlers to climb in vivo, and electrically adhesive hydrogels allow wheeled robots to climb on conductive substrates. However, these designs have inevitable defects. They can only climb on the surface of specific materials or only on the surface of specific structures. Therefore, it is an important challenge to develop climbing robots that adapt to complex surfaces (complex structures and complex materials), especially discontinuous surfaces.

In nature, *Parthenocissus* climbs as it grows (*19*), having growing-climbing behaviors. It can easily climb on complex surfaces, get into narrow gaps, and cross larger gaps by growing. A key factor that allows *Parthenocissus* to live on complex surfaces is the *Parthenocissus*’ suckers. There are many microstructures (*20*) on the *Parthenocissus*’ suckers to enhance adsorption. *Parthenocissus* will grow suckers and fix them on the wall and then grow their trunks upward. This method allows the already-grown parts to be firmly attached to the wall to avoid affecting subsequent growth. The *Parthenocissus*’ suckers are obtained by growth, and the suckers adhere to the support to promote the growing and climbing of the *Parthenocissus*. In addition, *Parthenocissus* may complete hinge-like bending through the contraction of the gelatinous fibers on one side (*21*), thereby ensuring that the position of the bending area does not change over time. So, can the way *Parthenocissus* grows, climbs, and bends be applied to climbing robots? Can climbing robots designed on the basis of this method further verify the way *Parthenocissus* grows, climbs, and bends?

In the field of soft robots, a class of growing robots (*22*–*24*) that grow like *Parthenocissus* has attracted a lot of attention. However, existing growing robots cannot achieve the growing and climbing of *Parthenocissus*. First, the current growing robots grow freely and cannot climb. Second, the continuity of the growing robot brings various challenges to its bending, and the poor bending performance cannot adapt to nonstructured environments. There are some existing methods to achieve bending of growing robots. The internal controllable catheter (*25*) controls the bending direction of the growing robot by pulling the tendon connected to it. It can be applied to a growing robot of millimeter size, but it cannot achieve continuous multiple turns. Series pneumatic artificial muscles (sPAMs) (*26*–*28*) and fabric pneumatic artificial muscle (*29*) are typical bending methods of growing robots. By installing additional pneumatic muscles on the growing robot to apply tension to the robot’s growing body, a bending with a nearly constant curvature can be achieved. However, the pneumatic muscle is controlled by a gas source and cannot accurately control multiple turns. Cylindrical pneumatic artificial muscles (*30*) divide sPAM into segments and control it with multiple valves, which can achieve multiple active turns. However, the added structure will increase the weight and the bending angle is limited. Embedding electromagnets inside the growing robot can achieve accurate bending, locking, and release, but because of the heavy weight of the electromagnets, the weight of the growing robot will increase substantially. Adding bending mechanisms [skeleton (*31*) and tip steering mechanism (*32*, *33*)] inside the growing robot can achieve accurate bending, giving the soft growing robot continuous high curvature bending ability, but the weight of the growing robot will also increase substantially. Hot welding (*34*) and three-dimensional (3D) printing (*22*) during the growth of the robot can generate the necessary bending points in real time, but this bending is one time and cannot be changed. The bending of the soft climbing robot should be large angle, fast response, and controllable, and the components that enable bending should be lightweight. By going one step further than *Parthenocissus*, when the task is completed, the growing robot should be able to contract so as not to cause damage to the site. There are two main existing contraction mechanisms, one is the tip contraction mechanism (*35*) and the other is the self-contracting mechanism (*36*), but both methods cannot take into account lightness and low complexity. The growing robot used to achieve complex surface climbing should have the ability to grow, climb, bend quickly and controllably, and contract simply. Therefore, it is extremely challenging to develop a class of growing robots suitable for climbing complex surfaces, especially discontinuous surfaces.

**Fig. 1.**
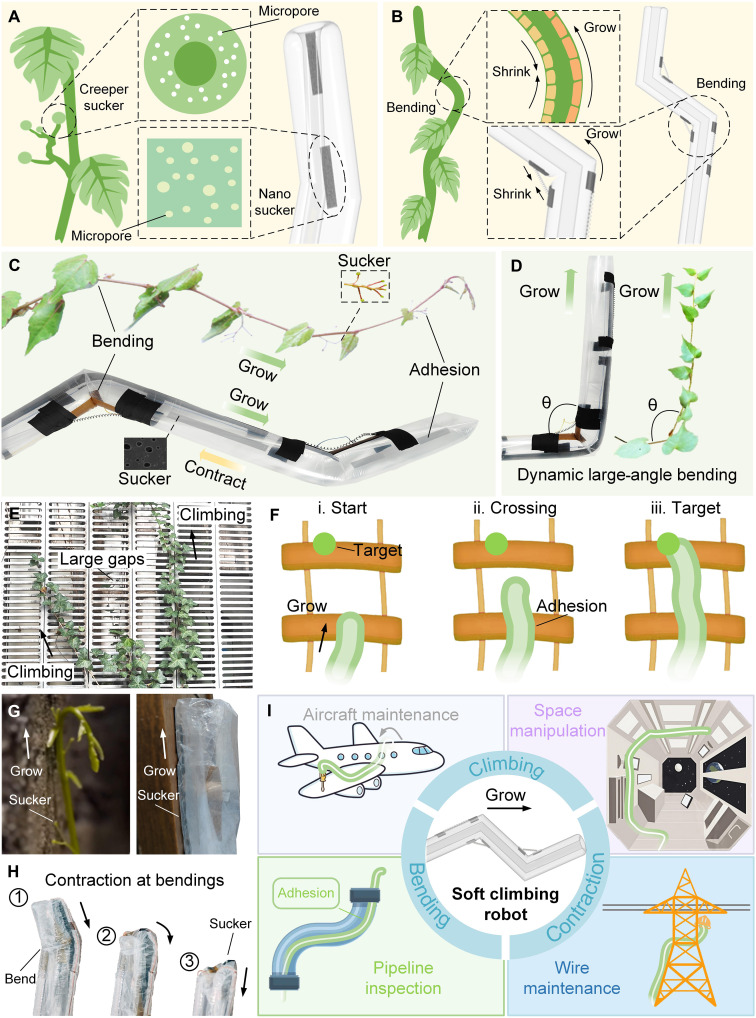
Concept of the *Parthenocissus*-inspired soft climbing robot. (**A**) The suckers of *Parthenocissus* and the robot. There are micropores on the suckers. (**B**) The bending of *Parthenocissus* and the robot. Contraction on one side causes bending. (**C** and **D**) Dynamic large-angle bending. The bending angles of *Parthenocissus* and the robot can be small or large. (**E**) Climbing plants climbing on discontinuous surfaces. There are large gaps on discontinuous surfaces (**F**) Soft climbing robot climbing on discontinuous surfaces. The robot crosses the large and reaches the target. (**G**) Growing and climbing of *Parthenocissus* and the soft climbing robot. (**H**) Contraction of the soft climbing robot. The bending can also be contracted. (**I**) Applications of the soft climbing robot.

Here, inspired by the growing and climbing of *Parthenocissus*, we propose a climbing robot locomotion mechanism, the growing-climbing mechanism. The growing-climbing mechanism increases adhesion through growing and promotes climbing through adhesion, thereby achieving climbing on complex surfaces, especially discontinuous surfaces. On the basis of the growing-climbing mechanism, we create a *Parthenocissus*-inspired soft climbing robot (diameter, 5.8 cm; growing length can be customized according to different tasks) (Fig. 1A and fig. S1). The growth of *Parthenocissus* is driven by cell turgor pressure, while the growth of the soft climbing robot is driven by a pneumatic system (fig. S2). *Parthenocissus* climbing depends on suckers, while the robot climbing depends on microstructured biofilms (width, 2.5 cm; thickness, 0.5 mm) as its suckers; the suckers of *Parthenocissus* are grown, while the microstructured biofilms of the robot are grown by material eversion; the bending of *Parthenocissus* is achieved by the contraction of gelatinous fibers (Fig. 1B), while the bending (maximum, 80°) of the robot (Fig. 1, C and D) is achieved by the contraction of shape memory alloy (SMA) springs (the length is 15 cm and 1.5 cm when heated, installed on the outside of the robot, with intervals greater than 2 cm). One step further than *Parthenocissus* is that when the robot completes its task, it can also completely contract its body and suckers through the contraction of the growth material (fig. S1) and the adhesion of the biofilm to the wall. The growing-climbing mechanism allows the robot to climb on complex surfaces, especially discontinuous surfaces (Fig. 1, E and F). The grown part firmly grasps the wall (Fig. 1G), thus avoiding drift problems during bending and buckling problems during contraction (Fig. 1H). Compared with existing bending and contraction mechanisms (*27*, *35*–*37*), our bending mechanism is large angle, fast response, controllable, and lightweight, and our contraction mechanism is lightweight and easy to manufacture. The proposed growing-climbing mechanism solves the important challenge of adapting to complex surfaces, especially discontinuous surfaces, greatly expands the possibilities of climbing robots (Fig. 1I), and verifies the effectiveness of the growing-climbing mechanism and bending mechanism of the *Parthenocissus*.

## RESULTS

### Growing-climbing mechanism

**Fig. 2.**
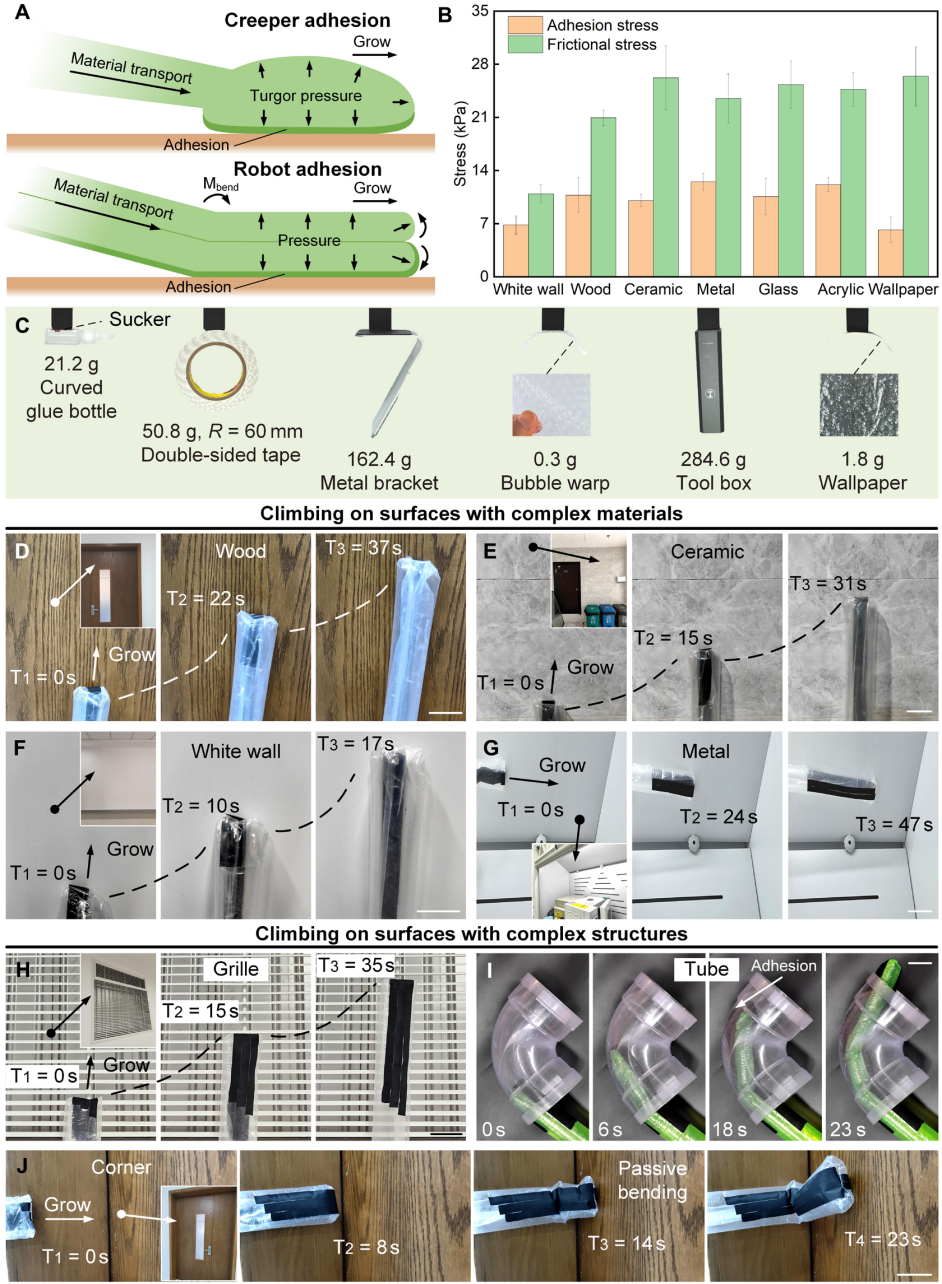
The growing-climbing mechanism of the soft climbing robot. (**A**) The principle of the growing-climbing mechanism, including driving force, material transport, and adhesion. (**B**) The adhesion stress and friction stress of the microstructured biofilms. (**C**) Demonstration of the adhesion of the microstructured biofilms to various objects with different weights, sizes, and materials. (**D** to **G**) Soft climbing robot climbing on surfaces of different materials, including wood, ceramic, white wall, and metal (movie S1). (**H** to **J**) Soft climbing robot climbing on surfaces with complex structures, including the grille, the tube, and the corner (movie S2). Scale bars, 5 cm.

*Parthenocissus* grows to climb and climbs to grow better. The growing-climbing mechanism of *Parthenocissus* includes driving force, material transport, and adhesion (Fig. 2A). The driving force comes from cell turgor pressure, which is the pressure of water in the cell on the cell wall. Cell turgor pressure causes the deformation of the cell wall at the growing tip of *Parthenocissus*, thereby promoting cell expansion and growth of *Parthenocissus*. The raw materials for the growth of *Parthenocissus* depend on the material transport in the body. Nutrients are transported to the tip to synthesize the cell wall. The reason why *Parthenocissus* can climb on various complex surfaces is its sucker, which has many microstructures for adsorbing to the wall. The growing and climbing of *Parthenocissus* are inseparable. When *Parthenocissus* climbs, the internal cell turgor pressure provides the driving force, the nutrients at the distal end are transported to the tip, and new suckers are formed. At the same time, because of the internal pressure, the sucker is firmly adsorbed to the wall. After having an attachment point, *Parthenocissus* continues to grow upward.

Inspired by the growing-climbing mechanism of *Parthenocissus*, we create a soft climbing robot that combines growing and climbing (Fig. 2A) (see “Fabrication and materials of the soft climbing robot” in Materials and Methods). The growing-climbing principle also includes driving force, material transport, and adhesion. The driving force of the soft climbing robot is air pressure, and the eversion of the growth material participates in the growth of the tip as a way of material transport (fig. S1). Imitating the sucker of *Parthenocissus*, microstructured biofilms (figs. S3 and S4, see “The microstructured biofilms” in Materials and Methods) are added to the soft climbing robot. The biofilm is a flexible sucker that uses van der Waals force and negative pressure effect to generate adhesion. The microstructured biofilm is covered on the growth material. In the ungrown state (fig. S1B), the growth material with the microstructured biofilm is contracted inside, and when inflated, the internal pressure will cause the growth materials to stick together tightly. Therefore, to prevent the biofilms from adhering to each other and thus blocking growth, the biofilm is placed on one side of the growth material, while a nonadhesive material (e.g., felt) is placed on the other side to prevent the internal materials from adhering to each other. During the growing process, while the eversion of the growth material causes the tip to grow, the microstructured biofilm is also everted and adheres to the wall. After having an attachment point, the soft climbing robot continues to grow upward.

We build a physical model of growing-climbing, which mainly includes a growing model and a tip adhesion model caused by the eversion of the adhesion material. The growing model includes three items: plant cell extension model, path loss model, and tip peeling model

$$\mathrm{PA}=\left[\mathrm{YA}+{\left(\frac{1}{\varphi }v\right)}^{\frac{1}{n}}A\right]+\left[\mu_{s}\mathrm{wL}+\sum_{i}Ce^{\frac{\mu_{c}L_{i}}{R_{i}}}\right]+\frac{\mathrm{tG}}{1-\text{cos}\theta }$$ (1)

where *P* is the driving pressure, *Y* is the minimum growing pressure, φ is the extensibility, *v* is the tip velocity, *n* is a power term close to 1, *A* is the cross-sectional area of the eversion point, *C* is a coefficient based on exponential fitting, μ*_c_* is the friction coefficient due to curvature, *R* is the radius of curvature, *L* is the length of the soft robot path, μ*_s_* is the length-dependent friction coefficient, *w* is the normal force applied per unit length, *t* is the width of the adhesion layer, and *G* is the crack energy of interface fracture per unit area. Because of the presence of the adhesive material, the inner material is tightly adhered together, and when the material grows outward, there will be an eversion peeling force. According to the formula, when the driving pressure remains unchanged, the smaller the peeling force, the greater the growing rate. Therefore, we cover the surface of the other side of the same position of the adhesive material with a layer of nylon material with lower surface energy to reduce the peeling force. The tip adhesion model mainly includes the van der Waals force model and the negative pressure adsorption model

$$F_{\mathrm{adh}}=F_{\text{vdw}}+A\Delta P$$ (2)

where *F_adh_* is the adsorption force, *F_vdw_* is the van der Waals force, *A* is the contact area, and Δ*P* is the pressure difference. The internal pressure and the bending stiffness of the grown part make the microstructured biofilm interact with the contact surface. *F_vdw_* and A in the formula increase, thereby enhancing the adhesion strength.

We test the adsorption strength of biofilms on different surfaces (Fig. 2B), including common walls (white wall, wood, ceramic, metal, glass, acrylic, and wallpaper). The adhesion stress of the biofilm on smooth surfaces (e.g., ceramic, metal, glass, and acrylic) can reach 10 kPa, which is better than the adsorption performance on rough surfaces (e.g., white wall and wood). The adhesion stress on the rough surface can reach 7 kPa. At the same time, the friction stress of the biomimetic membrane is stronger than the adhesion stress. Taking the ceramic surface as an example, the friction stress can reach 26.2 ± 3.2 kPa, while the adhesion stress can only reach 10.1 ± 0.8 kPa. Because of the excellent contact adaptability of the biofilm, the biofilm is suitable for lifting objects of various weights, sizes, materials, and roughness (Fig. 2C). Specifically, the surfaces in the experiments include flat metal surfaces [e.g., toolbox (weight, 284.6 g; contact area, 9 cm^2^) and metal bracket (weight, 162.4 g; contact area, 9 cm^2^)], curved surfaces [e.g., curve glue bottle (weight, 21.2 g; contact area, 2.4 cm^2^), double-sided tape (radius, 60 mm; weight, 50.8 g; contact area, 2 cm^2^)], and rough surfaces [e.g., bubble warp (weight, 0.3 g; contact area, 4.71 cm^2^) and wallpaper (weight, 1.8 g; contact area, 9 cm^2^)]. When the soft climbing robot integrated with microstructured biofilm climbs on a vertical metal wall, the maximum load can reach about 1330 g/cm^2^.

To demonstrate the climbing ability of the soft climbing robot on complex surfaces, we conducted climbing tests on surfaces with complex materials and complex structures. The complex material surfaces include wood, ceramic, white wall, and metal (Fig. 2, D to G, and movie S1). It has been proven that the soft climbing robot has good climbing performance on many common material surfaces. Complex structures include discontinuous surfaces, inside a tube and a corner (Fig. 2, H to J, movie S2). When the soft climbing robot climbs a discontinuous surface, its adhesion makes the grown part firmly adhere to the grid, and its growability enables it to cross a larger gap and find the next attachment point. In the tube climbing test, the soft climbing robot’s softness and adhesion make it tightly bonded to the inner walls of the tube. The robot is passively bent according to the shape of the tube and finally passes through the tube. In the corner test, the soft climbing robot can fill the 90° corner and pass smoothly because of its flexibility.

### Growing-bending mechanism

**Fig. 3.**
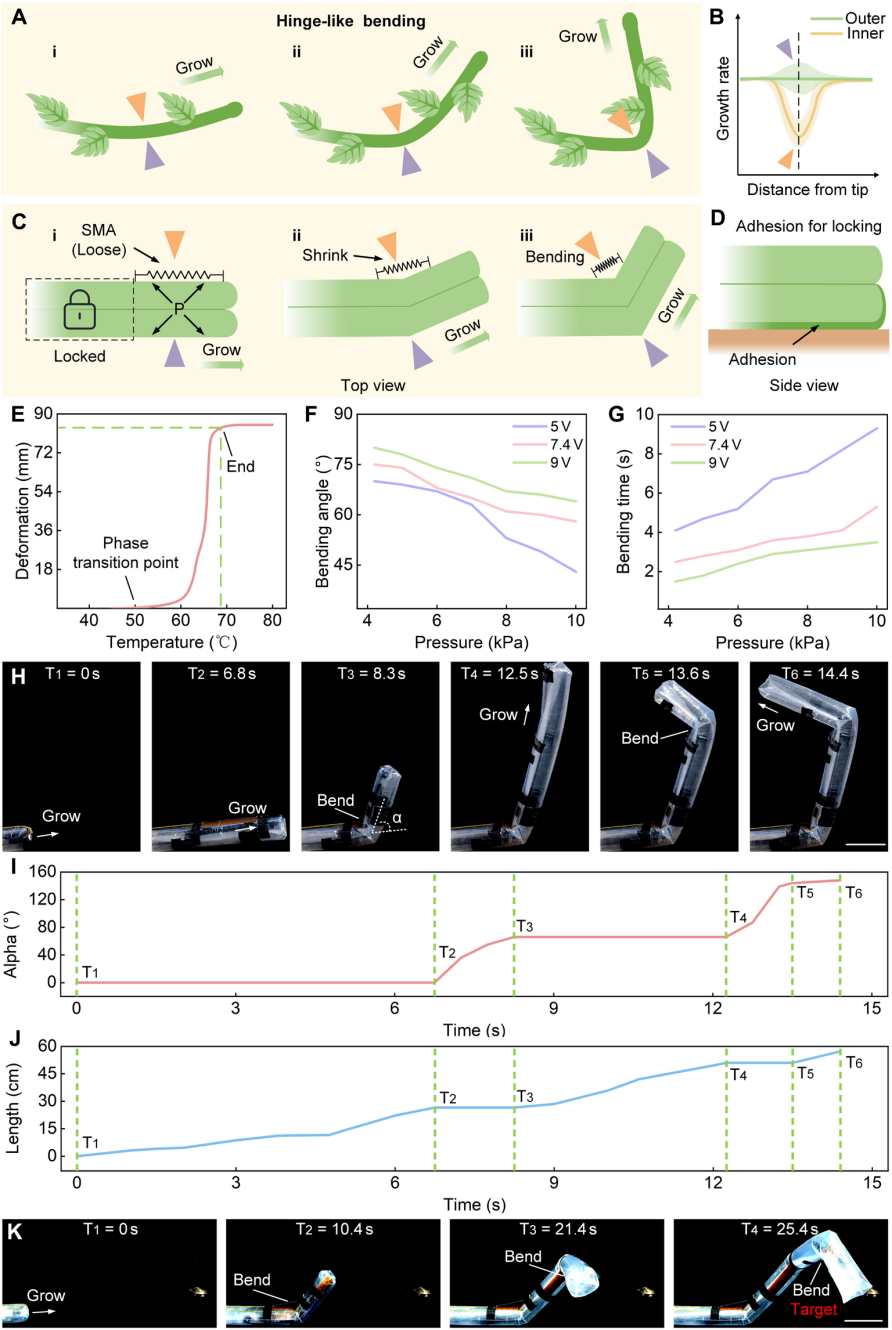
The growing-bending mechanism of the soft climbing robot. (**A**) The hinge-like bending of *Parthenocissus.* The bending position does not change with time. (**B**) Growth rate of the inner and outer sides of the bend. The inner growth rate is slower than the outer growth rate (**C** and **D**). The principle of the growing-bending mechanism. The parts that have grown and bent are locked. (**E**) Deformation test of SMA spring. (**F**) Test of the robot’s bending angle under different pressures. (**G**) Test of the robot’s bending time under different pressures. (**H** to **J**) Continuous bending of the soft climbing robot. The robot undergoes two bends, and the angle α and the growing length are recorded. α is defined as the angle between the robot head axis and the horizontal line (movie S3). (**K**) 3D growth of the soft climbing robot (movie S4). Scale bars, 10 cm.

Macroscopically, the growing-bending process of *Parthenocissus* can be classified as hinge-like bending according to its shape (Fig. 3A), that is, the position of the bend does not change over time (Fig. 3B) and is firmly fixed to the wall because of the sucker. Microscopically, the growing-bending mechanism of *Parthenocissus* involves the contraction of the gelatinous fibers, which are contained between the epidermis and the vascular column of the vine. During the bending process, these gelatinous fibers undergo gel-like contraction. The growing-bending mechanism of *Parthenocissus* is of great significance for climbing and bending behaviors, ensuring that the parts that have grown and bent and the parts that will grow and bend do not affect each other. Inspired by the growing-bending mechanism of *Parthenocissus*, the growing-bending of the soft climbing robot follows three principles: first, hinge-like bending; second, the parts that have grown and bent are locked without affecting subsequent growth; and third, growing-bending involves contraction on one side. The soft climbing robot generates wrinkles on one side, causing the overlap of the growth material and resulting in the length of this side being shorter than the length of the other side (Fig. 3C). The driving force for the wrinkles is the phase transition of the SMA spring. When the power is on, because of the thermal effect, the SMA spring shortens rapidly, causing the robot to bend. After the power is turned off, the internal force of the spring still exists and can resist the tension of the air pressure in the main air chamber, so the state of the robot after bending can be maintained for a certain period of time. When the temperature cools down, the SMA spring can be stretched again. However, because of the existence of the sucker, the grown and bent part is firmly fixed to the wall and maintains its shape (Fig. 3D).

The SMA spring used can be stretched to 15 cm, rebound to 1.5 cm when heated, and the deformation can reach 13.5 cm. When the temperature rises to 50°C, the SMA begins to undergo a phase change, and when the temperature reaches 68°C, the SMA spring is completely contracted (Fig. 3E). The SMA spring is integrated into the robot, and the bending performance is tested. Before the experiment, the spring is stretched to 15 cm. Three voltages are applied in the experiment to test the contraction time, length change, and bending angle of the robot under different pressures. According to the data (Fig. 3, F and G), it can be seen that as the driving pressure decreases, the bending angle gradually increases, and at 4.2 kPa, the bending angle can reach 80° (voltage, 9 V). At the same time, as the internal pressure decreases, the bending response time also gradually decreases and can reach 1.5 s at 4.2 kPa (voltage, 9 V). In addition, as the voltage increases, the bending angle becomes larger and the bending time decreases.

The growing and bending process of the soft climbing robot is shown in Fig. 3 (H to J) and movie S3. At T_1_, the soft climbing robot is in the initial state and grows forward. At T_2_, the power supply at both ends of the SMA spring is turned on, and the spring contracts and drives the robot to turn left (the bending angle is about 66°). After turning, the robot can continue to grow forward while maintaining its original state. At T_4_, the robot repeats the previous bending process, continues to grow forward (T_5_), and eventually forms the motion state shown at time T_6_. During the whole process, the motion state of the robot’s grown part can remain in place and is not affected by the robot’s subsequent motion process. In addition, the soft climbing robot can perform complex 3D growth and reach the target after three turns (Fig. 3K and movie S4).

### Contraction mechanism

The contraction of growing robots is an ongoing challenge. *Parthenocissus* climbs on high walls to get sunlight through the growing-climbing mechanism and does not need to consider how to contract its body. One step further than *Parthenocissus* is that after completing the task, the soft climbing robot needs to consider how to contract its body to avoid damaging the site. The basic implementation idea is to achieve the reverse movement of the robot’s growth or, more precisely, to pull the grown part of the robot material back in the opposite direction through its internal materials. As the dc motor turns, the material contracts, and the soft climbing robot contracts (fig. S1).

**Fig. 4.**
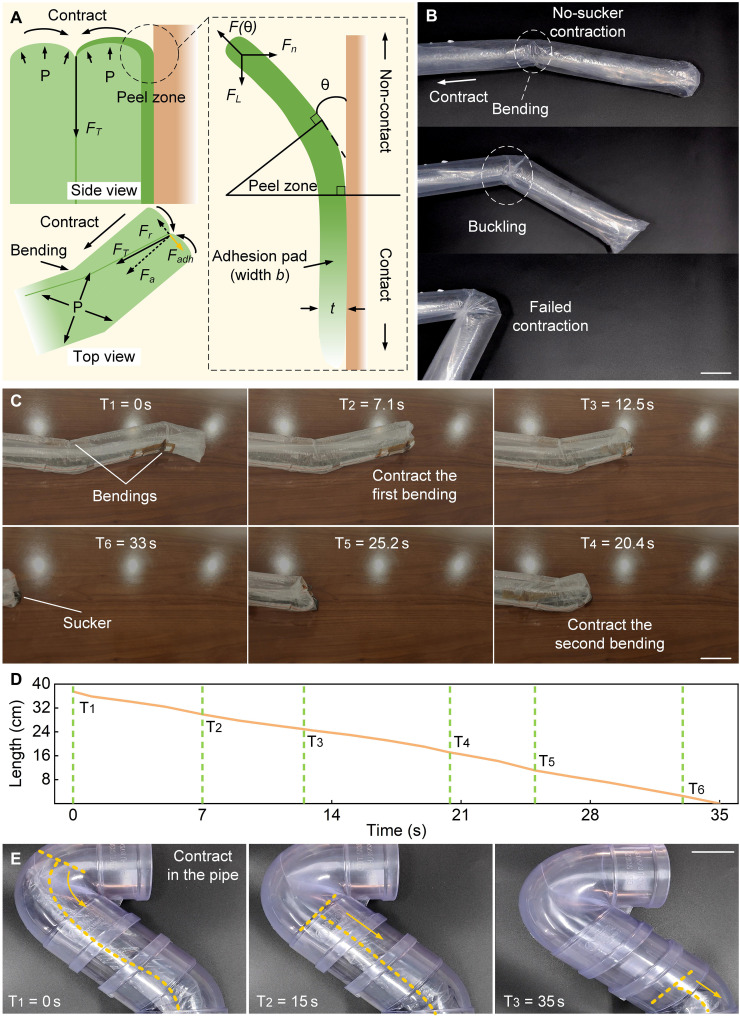
The contraction mechanism of the soft climbing robot. (**A**) The principle of the contraction mechanism. The contraction of the robot can be considered as a peeling process. (**B**) Growing robots without additional contraction devices will buckle. (**C** and **D**) The contraction process of the continuous bending soft climbing robot (movie S5). The length of the contraction varies linearly with time. (**E**) The contraction process of the continuous bending soft climbing robot inside a pipe. The robot is peeled along the curved pipe wall. Scale bars, 5 cm.

The reverse movement of the robot’s growth when the robot is in a bending state is shown in Fig. 4A. During contraction, the tension generated by the internal material is not axial but has a certain angle with the robot axis (see Fig. 4A, top view). This angle changes with the bending angle of the robot. We can decompose the tension *F* of the internal material into axial force *F_a_* and radial force *F_r_*. The axial force *F_a_* can pull the robot back axially. We analyze the force of the robot’s tip. Let the angle between the component force and the thin wire be α. At this time, the air pressure applied inside the robot is *P*, and the diameter of the robot is *d*. To contract the robot, the axial force generated on the thin wire must be greater than *F*, that is,

$$F_{a}=F\text{cos}\alpha >P\frac{\pi d^{2}}{4}$$ (3)

Since the material of the traditional growing robot is only a layer of polyethylene (PE) film, the radial force that the robot can withstand is very weak. When the radial force *F_r_* acts on the robot in the radial direction, the robot will bend under the action of traction because it cannot withstand the force of the thin wire in the vertical direction (Fig. 4B). As the bending angle increases, the robot will form a tangled phenomenon, making it difficult to contract. Therefore, to keep the robot in place during the contraction, the following formula should be satisfied

$$F_{r}<F_{\mathrm{adh}}$$ (4)

The fundamental reason why the growing robot is difficult to contract is that its radial bearing capacity is too weak to support the radial force. Because of the adhesion effect of the sucker, there is a strong adhesion force *F_adh_* between the soft climbing robot and the wall. The adhesion force *F_adh_* offsets the effect of the radial force *F_r_* and solves the problem of the contraction of the growing robot. Considering the interaction between the soft climbing robot and the wall, the contraction can be converted into a peeling behavior similar to the bioadhesion system (*38*), which can be described by the Kendall model

$$F=\frac{\mathrm{tG}}{1-\text{cos}\;\theta }$$ (5)

where *F* is the peeling force, θ is the peeling angle, *G* is the crack energy per unit area of the interface fracture, and *t* is the width of the adhesion layer. The Kendall model describes the relationship between the peeling strength and the peeling angle. The larger the peeling angle, the smaller the peeling force. The peeling angle of the soft climbing robot can be approximated to 180°.

To demonstrate the contraction performance of the soft climbing robot, we conducted a contraction experiment (movie S5). Before the contraction, the soft climbing robot has two bendings. When the robot contracts to the place where the biofilm is attached, the biofilm will be peeled off on the plane in the form of tearing tape, and then the remaining part will slowly leave the wall as the robot contracts. The soft climbing robot completes the contraction at the first bending and the second bending at T_2_ and T_5_, respectively (Fig. 4D). In addition, the soft climbing robot can also rely on this method to complete contraction in a curved narrow pipe (Fig. 4E).

### Applications

**Fig. 5.**
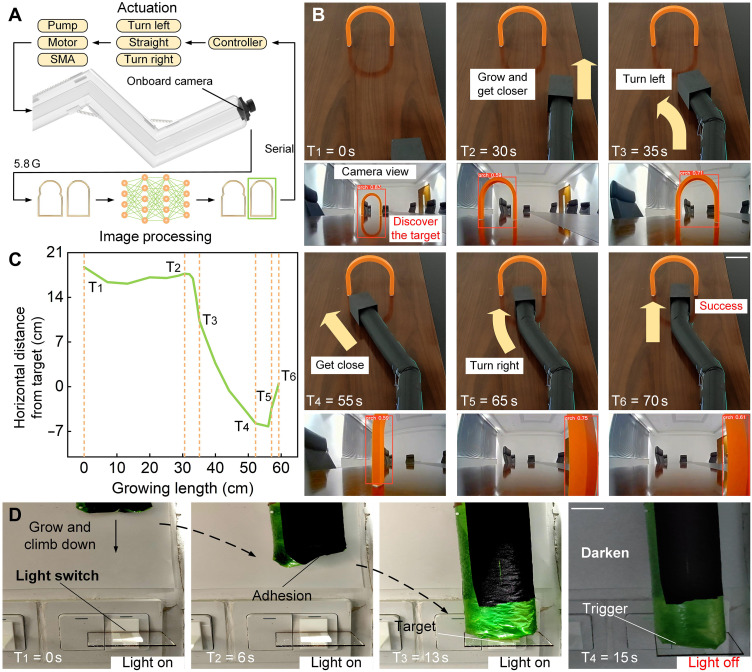
Soft climbing robot for visual inspection. (**A**) Software and hardware framework for soft climbing robot vision inspection, including actuation and image processing. (**B**) The soft climbing robot visually recognizes and passes through an arched door. The arch is identified from the camera’s view (movie S7). Scale bars, 5 cm. (**C**) The horizontal distance from the target during the growth. (**D**) The soft climbing robot turns off the lights. Robots can complete manipulation tasks through the power of growth. Scale bars, 2.5 cm.

In real environments, the soft climbing robot may encounter various unknown environmental obstacles. To improve the robot’s ability to operate in different environments, we use image processing technology (see “Object detection applications” in Materials and Methods) to perform real-time road condition analysis and path planning during the robot’s growing and climbing. A camera module is installed on the robot’s head (fig. S10) so that it can acquire image information during operation and return the image information to the terminal for display and processing. The camera module includes a camera and a video transmitter for transmitting videos. It also includes a battery and a boost board to power the camera and video transmitter. The soft climbing robot is in close contact with the side wall of the module, and the impact force of the growth pushes the camera module forward. The detected objects are classified by the target detection algorithm built by the neural network model. The position of the target in the image is confirmed and highlighted in the form of a label. Then, the feedback position information will be converted into command control for the robot to turn left, go straight, or turn right, so as to realize the normal operation of the robot in the actual environment. In the experiment, a structure similar to the shape of an arch is designed. Before the experiment, a neural network model is trained for the arch so that the robot can recognize the structure during operation (Fig. 5A). In the experiment, the camera detects the targets on the road in real time during the robot’s movement, and the robot is controlled to turn left or right accordingly (Fig. 5B and movie S7). The curve (Fig. 5C) shows the change of the horizontal distance of the robot from the center of the target with the growing length of the robot. As can be seen from Fig. 5C, the change in distance from the target position corresponds well to the steering control required at each position during the robot’s movement. In addition to visual observation, climbing robots are expected to perform manipulation tasks such as flipping switches or tightening valves. Typical climbing robots need to add additional actuators to complete the above tasks, but the soft climbing robot can complete these manipulation tasks through the power brought by growth. We tested the impact force of the growth (fig. S6) and construct a scenario where the soft climbing robot turns off the lights (Fig. 5D). The soft climbing robot grows and climbs from top to bottom. During the entire process, since the robot always adheres to the wall, the grown parts will not be affected by the growing parts and triggers. Last, the soft climbing robot reaches the target and turns off the lights through the force of its growth.

**Fig. 6.**
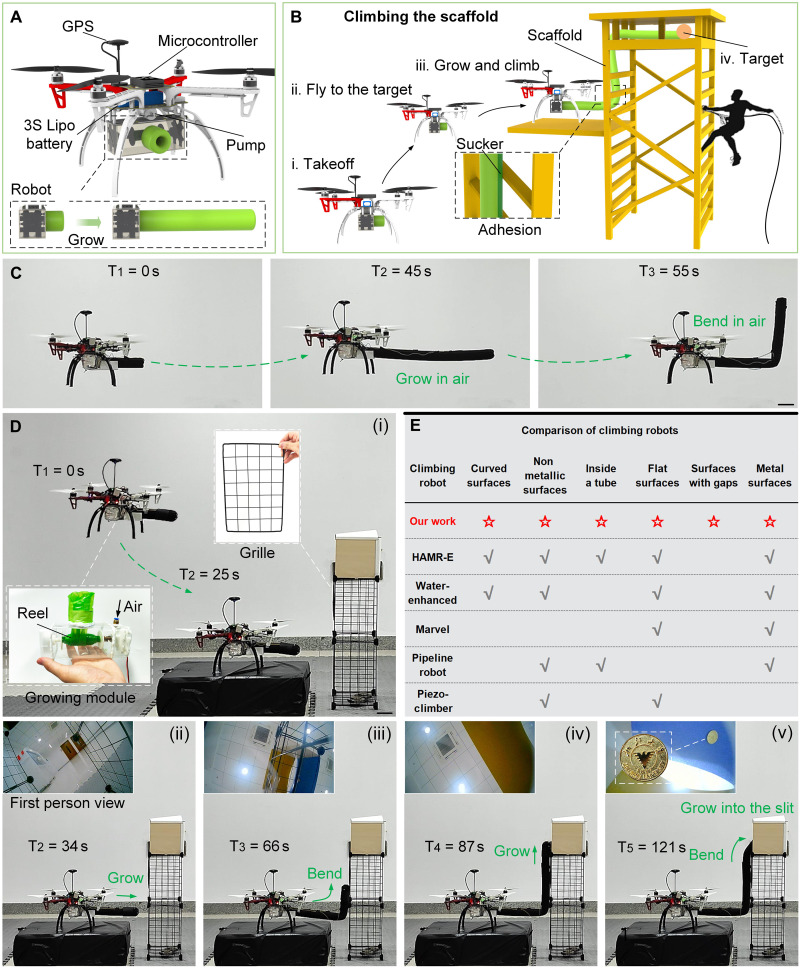
Soft climbing robot combined with a drone for construction site inspections. (**A**) A drone carrying a growing module, which is portable and integrated. (**B**) Imagined operation scenarios for the combination of a drone and a soft climbing robot to reduce human risks. (**C**) Aerial growth of the soft climbing robot (movie S8). (**D**) A soft climbing robot and a drone combined for 3D exploration. The first person view is from the camera at the front end of the soft climbing robot (movie S9). (**E**) Comparison with other climbing robots. Scale bars, 10 cm.

Soft climbing robots can also cooperate with other types of robots to complement each other’s strengths and gain a wider range of movement capabilities to complete more challenging tasks. There are a large number of discontinuous surfaces in scenes such as construction sites, mining, and production workshops. Workers usually need to climb scaffolding to complete their work, which poses certain risks. People expect to use climbing robots to avoid these risks, but conventional crawling robots cannot climb on the discontinuous surface of the scaffolding. The soft climbing robot can climb, bend, and contract on complex surfaces, especially discontinuous surfaces. It can be used as an extension of the drone (*39*, *40*) so that the entire system has the ability to fly and climb for 3D operations. For portability, a small and integrated growing module (weight, 284 g) is designed and installed on a quadcopter drone (Fig. 6A and fig. S12) and is powered and controlled by the drone (see “Soft climbing robot on the drone” in Materials and Methods). In the envisioned environment, the drone can fly to a certain layer of the scaffolding and stop there. Then, the soft climbing robot can grow out of the growing module and climb up the scaffolding, pass through the narrow gap, and reach the target point (Fig. 6B). It is worth mentioning that the soft climbing robot has the ability to grow in the air and bend at a large angle (Fig. 6C and movie S9). However, considering the stability of the entire system, when the soft climbing robot adheres to the surface, the interaction between them will cause the drone to lose balance, so we hope that the drone can be docked on a platform before operating. We built a miniature model of a construction site (Fig. 6D), including a small grille-shaped scaffolding and a platform. On the top floor of the scaffold, two wooden planks form a passage with a ceiling. There is a target that needs to be explored in the passage, which the drone cannot see directly. The drone carrying the growing module lands on the platform first. Then, the soft climbing robot with a camera on the front grows forward until it encounters the obstruction of the scaffold. After that, the soft climbing robot completes the first bend and climbs up along the scaffold until the camera is higher than the scaffold. At this point, it completes the second bend and continues to grow forward to see the target in the slit. The combination of drones and soft climbing robots is complementary. During operation, drones need to stay away from dangerous walls, while the climbing of soft climbing robots can interact with complex surfaces; drones can move in a large range, while soft climbing robots focus on small-scale reconnaissance such as slits. Compared with other climbing robots (*1*, *4*, *6*, *7*, *41*), the soft climbing robot has a wider range of climbing adaptability (Fig. 6E), especially climbing on discontinuous surfaces.

## DISCUSSION

Climbing on complex surfaces, especially discontinuous surfaces, is a constant challenge for robots. We summarize a growing-climbing mechanism that combines growing and climbing from the growing and climbing behavior of *Parthenocissus*, which is growing for climbing and climbing for growing. On the basis of this mechanism, we create a *Parthenocissus*-inspired soft climbing robot. It can climb on a wide range of complex surfaces, especially discontinuous surfaces, verifying the effectiveness of the growing-climbing mechanism of *Parthenocissus* and its adaptability to complex surfaces. We suggest a microstructured biofilm to enhance adhesion, similar to the suckers of *Parthenocissus* adsorbed on the wall; the microstructured biofilm is grown from the robot, similar to the suckers grown by *Parthenocissus*; we propose that SMAs shrink to achieve bending, similar to the hinge-like bending achieved by the shrinkage of gelatinous fibers in *Parthenocissus*; in addition, the soft climbing robot can be completely contracted after completing the task without damaging the site.

Different from the works that focus on the free growth of robots (*22*–*24*), the growing-climbing mechanism we summarized from the climbing behavior of *Parthenocissus* links growth and adhesion, allowing the climbing robot climb as it grows like *Parthenocissus*, opening up a previously unidentified path for climbing robots. Compared with other climbing mechanisms (*1*, *4*, *6*, *7*, *41*), the growing-climbing mechanism solves the major challenge that climbing robots cannot climb on complex surfaces, especially noncontinuous surfaces. Our work does not focus on growth itself; growth is just one of the technical points of climbing robots. Besides, our work solves the pain points of the existing growing robots’ insufficient bending and contraction performance. Compared with the existing bending mechanism (*22*, *25*–*34*) of growing robots, the SMA-based bending has the characteristics of large angle, fast response, controllability, and light weight. Compared with the existing contraction mechanism (*35*, *36*) of growing robots, the contraction mechanism based on the microstructured biofilm is light in weight and easy to manufacture and install.

In this study, we only used one adhesion scheme (microstructured biofilm adhesion) to verify the effectiveness of the growing-climbing mechanism. The adhesion scheme can also be replaced to achieve adhesion on other complex surfaces. The growing-climbing mechanism is a previously unknown and universal climbing robot paradigm that can expand the adaptability of climbing robots to an unprecedented range, thereby bursting out huge application potential. In addition, the growing-climbing mechanism can also be combined with other robots to promote complementary advantages and burst out new vitality.

## MATERIALS AND METHODS

### Fabrication and materials of the soft climbing robot

The material required for the robot to grow forward is rolled onto a reel, and the reel is placed in a sealed container. As the robot grows, the air pump continuously inputs air pressure into the container, and the pressure inside the container increases, causing the material at the outlet to evert. At the same time, the material rolled onto the reel is continuously output forward under the drive of pressure, thereby achieving the growth of the robot. The robot uses a 0.05-mm-thick PE film (Taobao, China) as the eversion material (fig. S1) and has a diameter of 5.8 cm after inflation. A 30 rpm speed dc motor (G12-N20, Handson Technology, China) is used for growth and contraction. Microstructured biofilms (Xiamen GBS Optronics, China) are used as suckers for soft climbing robots. The other side of the microstructured biofilm had an adhesive backing for bonding to the PE film surface. The pneumatic drive system (fig. S2) includes a pressure controller (ZES30AF, Qingwen, China), a metal oxide semiconductor field-effect transistor (maximum current, 20A), an air pump (130, Taobao, China), and a solenoid valve (voltage, 5 V).

### The microstructured biofilm

We used scanning electron microscopy to characterize the morphology of the microstructured biofilm (fig. S4). The surface of the microstructured biofilm has many micropores, like the suction cups of *Parthenocissus*, with diameters ranging from tens to hundreds of micrometers. When the microstructured biofilm comes into contact with a surface, each micropore is a tiny suction cup that can obtain a sufficiently large adsorption force, thereby tightly adsorbing the biofilm on the object. The microstructured biofilm uses van der Waals force for adsorption, which has the following advantages: (i) It can be reused, and its adsorption force will not weaken with the increase of the number of uses; (ii) no residue, after using the micro-absorption tape, no glue residue will be left, and the surface of the object will not be damaged; (iii) suitable for a variety of surfaces (Fig. 2, B and C); and (iv) appropriate adhesion strength. After testing, the microstructured biofilm can provide sufficient adhesion (>7 kPa) during the growth-climbing process of the soft climbing robot and will not cause internal adhesion due to excessive adhesion, blocking growth, or hindering contraction. Commonly used tapes are usually >100 kPa and can even reach 500 kPa (*42*), which can easily cause internal adhesion and block growth or hinder contraction. The disadvantage of the microstructured biofilm is that it cannot adhere to a surface that is too rough (e.g., felt), so we cover the felt on the surface of the growth body opposite to the location of the biofilm to avoid growth blockage caused by internal adhesion.

### Growth rate measurement

To better understand the effect of the pressure on the growth rate of the robot, the pressure input into the robot was changed and the growth rate of the robot under different pressure conditions was measured (fig. S5). As can be seen from the figure, as the pressure increases, the growth rate of the robot increases. In the experiment, a ruler was used to measure the length of growth, and the pneumatic drive system (fig. S2) was used to stabilize the pressure.

### Impact and compression tests

We used a push-pull force gauge (SH-200 N, HANDPI, China) to conduct five quantitative tests on the impact force and compression force (contact length, 10 cm; compression 1 cm) at different pressures (4.2 to 10 kPa) and took the average value (fig. S6 and movie S6). As the pressure increases, the impact force increases and can reach 10.1 N at 10 kPa. As the pressure increases, the compression force increases and can reach 23.2 N at 10 kPa. The experiment proves that the robot has a certain impact and compression capability. On the basis of this performance characteristic of the robot, it can be applied to some scenes in life where the force is small, such as home lights and door switches. At the same time, robots can also be used to bear weight, such as lifting heavy objects that are difficult to lift.

### The structure for bending

The SMA used is a nickel-titanium alloy wire spring (Guojia SMA, China) with a one-way memory effect. The SMA spring can be regarded as a resistor. The SMA spring has the ability to deform because the material temperature changes during the deformation process, resulting in a thermoelastic martensitic phase transformation inside. In the experiment, the temperature of the spring is generally changed by energizing. When the power is turned on, the SMA spring will shrink rapidly and the temperature generated may reach tens or even hundreds of degrees Celsius. The material of the robot body is a very thin PE film, whose main component is PE, which does not have the ability to withstand high temperatures. Therefore, if the spring is energized for a long time, the high temperature generated will melt the outer membrane of the robot. To solve this problem, we use glass fiber to isolate the SMA spring from the outer membrane of the robot’s main air chamber (fig. S1) so that the high temperature does not directly contact the PE film. The glass fiber can withstand temperatures above 300°C. At the same time, we design a fixing block to fix the spring, and its two ends are the positive and negative poles of the power supply. Then, the spring is stretched to a certain length and installed on the robot. In addition, considering that the placement density of SMA springs will affect the growth and contraction performance, we conducted an SMA spring placement interval experiment (fig. S7). As the SMA spring’s placement interval (the distance from one’s tail to one’s head) decreases, the growth rate decreases. The minimum interval of SMA is 2 cm (growing rate, 1.5 cm/s); otherwise, growth blockage will occur.

### Object detection applications

#### Overview

The soft climbing robot has SMA springs on the sides for steering, and a camera module is placed on the head, which includes a camera, a video transmitter, a battery, and a booster board. The pneumatic drive system (fig. S2) is used to stabilize the pressure of the soft climbing robot. An Arduino microcontroller is used as a controller to control the encoder motor and SMA springs to achieve left, straight, and right turns of the soft climbing robot. The YOLOv5 visual detection model runs on the host computer (Jetson orin NX, NVIDIA, USA) to achieve real-time image processing. During the whole process, the camera on the head of the soft climbing robot acquires images and transmits them back to the host computer through the video transmitter. The host computer processes the images and transmits instructions to the microcontroller through serial communication to achieve controllable movement.

#### Image processing

The host computer can get the position of the target in the image in real time by running the YOLOv5 target detection algorithm. By calculating the coordinates of the center point of the target, the host computer determines the direction of the target and transmits the instructions of going straight, turning left or right to the microcontroller through serial communication.

#### Steering control

The instructions from the host computer will be transmitted to the microcontroller in real time, using a bang-bang heading controller. However, before steering control, the microcontroller needs to know the eversion of the SMA springs. In this experiment, an encoder motor is used to feedback the motor position information. To accurately understand the status information of each spring based on the motor parameters, it is necessary to know the corresponding relationship between the robot movement distance and the spring position. Figure S10 introduces the segmentation rule of SMA springs. S_1_, S_2_, and S_3_ represent three memory alloy springs, and L_1_, L_2_, and L_3_ represent the length of the rightmost end of each spring from the set initial position. By debugging the microcontroller and the encoder motor, the robot length value and the corresponding pulse number when each spring is completely everted are recorded. When the value is greater than L_1_, spring S_1_ can shrink normally; when the value is greater than L_2_, spring S_2_ can shrink normally; and when the value is greater than L_3_, S_3_ can shrink normally. Moreover, only the SMA spring closest to the tip of the robot can be activated. After defining the rules, the microcontroller determines whether to execute the instructions from the host computer according to the rules and controls the motor and SMA springs through different serial ports. Considering that the SMA spring will continue to heat up and cause the problem of over temperature when powered on for a long time, a time function is introduced to disconnect the spring after it reaches the fully contracted state 2 s after being powered on.

### Soft climbing robot on the drone

The growing module is actuated by an external pump module (fig. S2), which is used to stabilize the pressure in the growing module and is powered by the drone. The dc motor in the growing module is connected to the drone and is controlled by the flight controller to achieve growth and contraction. In addition, the SMA springs used for bending are powered by the drone and triggered by the flight controller. All actions of the growing module (including growth, recovery, and bending) can be remotely controlled through the radio control (RC) receiver and telemetry connected to the flight controller (fig. S12).

## Supplementary Materials

### The PDF file includes:

Figs. S1 to S12 Table S1 Legends for movies S1 to S9 References
