# Supplementary material for: Parthenocissus-inspired soft climbing robots
Source: Sci Adv. 2025 Mar 26;11(13):eadt9284. doi: 10.1126/sciadv.adt9284 (PMC11939062; doi:10.1126/sciadv.adt9284)
Supplement: Supplementary file 1 — Figs. S1 to S12 Table S1 Legends for movies S1 to S9 References [file sciadv.adt9284_sm.pdf]

Science Advances
AAAS

Supplementary Materials for

Parthenocissus-inspired soft climbing robots

Kecheng Qin *et al.*

Corresponding author: Wei Tang, weitang@zju.edu.cn; Jun Zou, junzou@zju.edu.cn

Sci. Adv. **11**, eadt9284 (2025)
DOI: 10.1126/sciadv.adt9284

The PDF file includes:

Figs. S1 to S12
Table S1
Legends for movies S1 to S9
References

Other Supplementary Material for this manuscript includes the following:

Movies S1 to S9

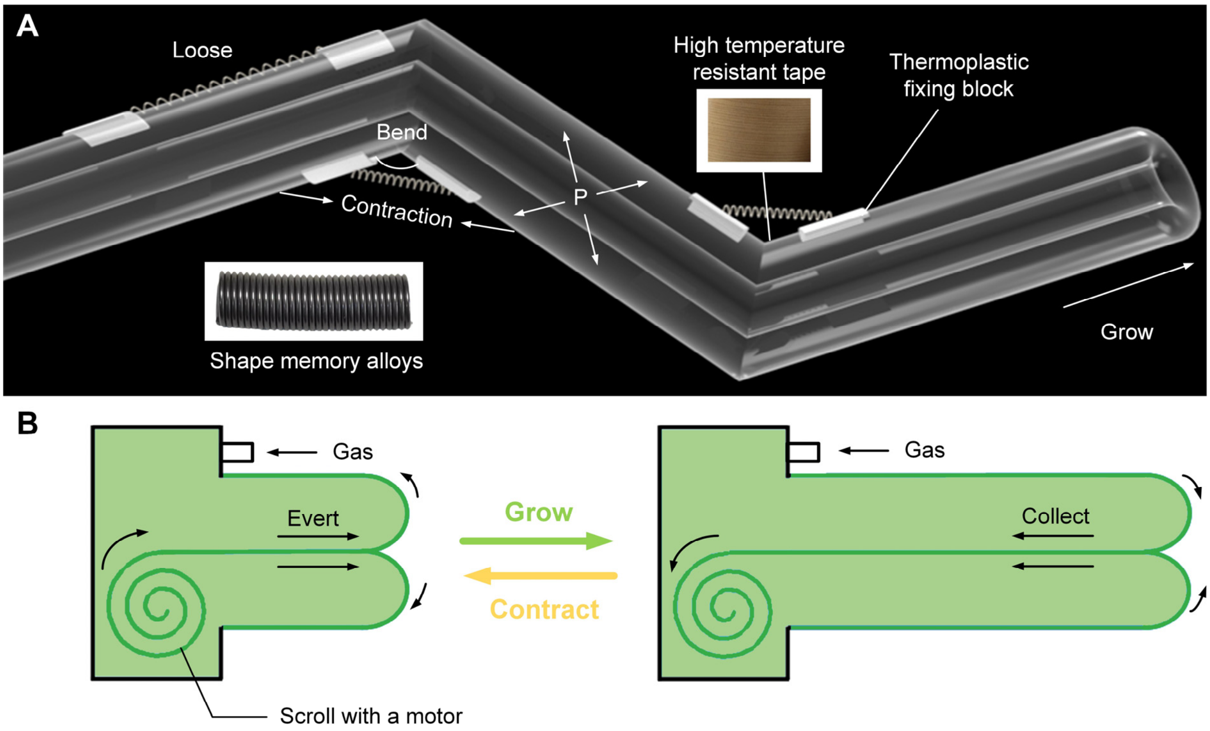

Fig. S1. Schematic diagram of the soft climbing robot. (A) Rendering of the soft climbing robot bending continuously. (B) The growth and contraction principle of the soft climbing robot.

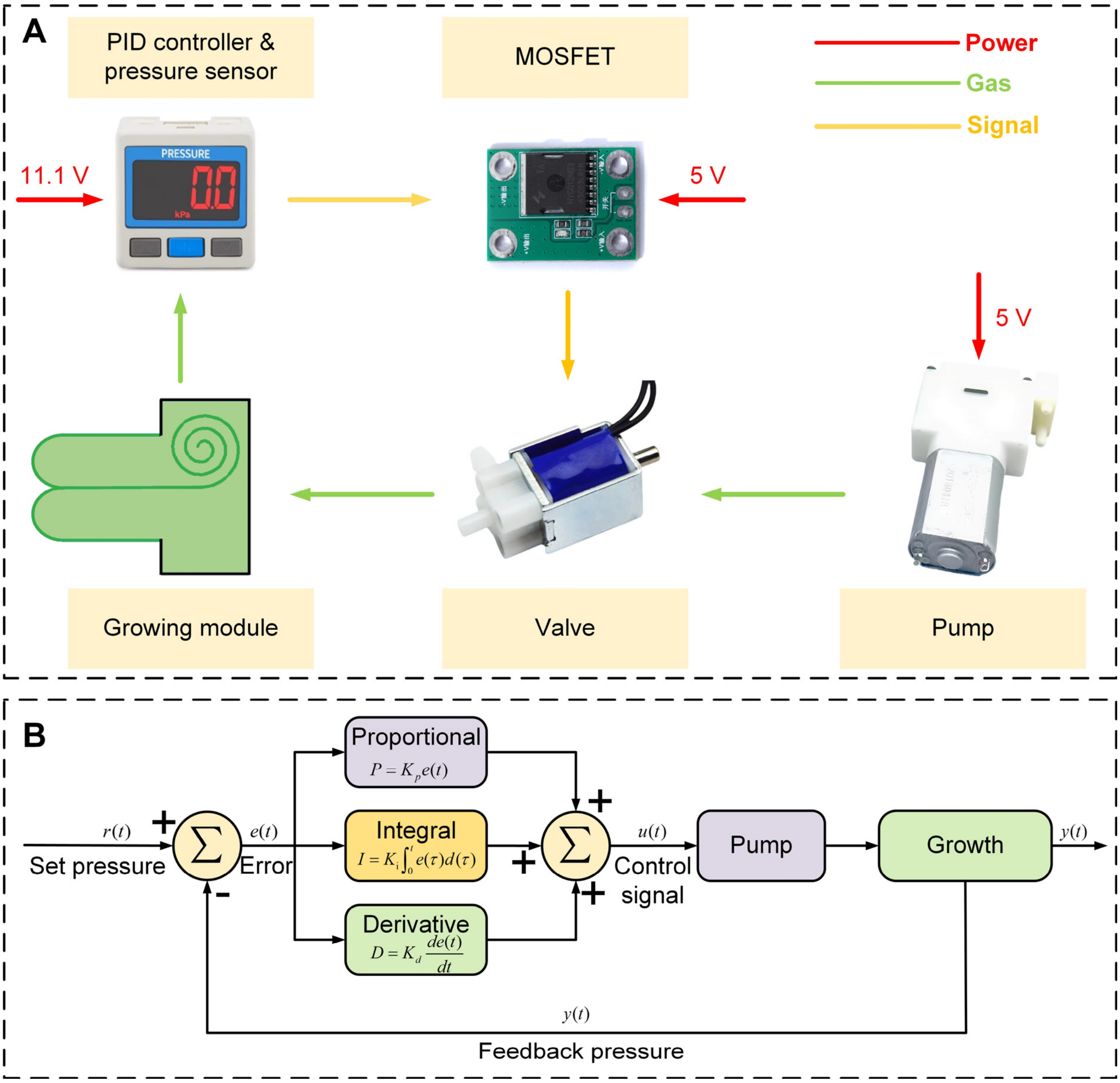

Fig. S2. Pneumatic drive system. (A) Composition and connection of the pneumatic drive system. (B) PID control algorithm for stabilizing the pressure.

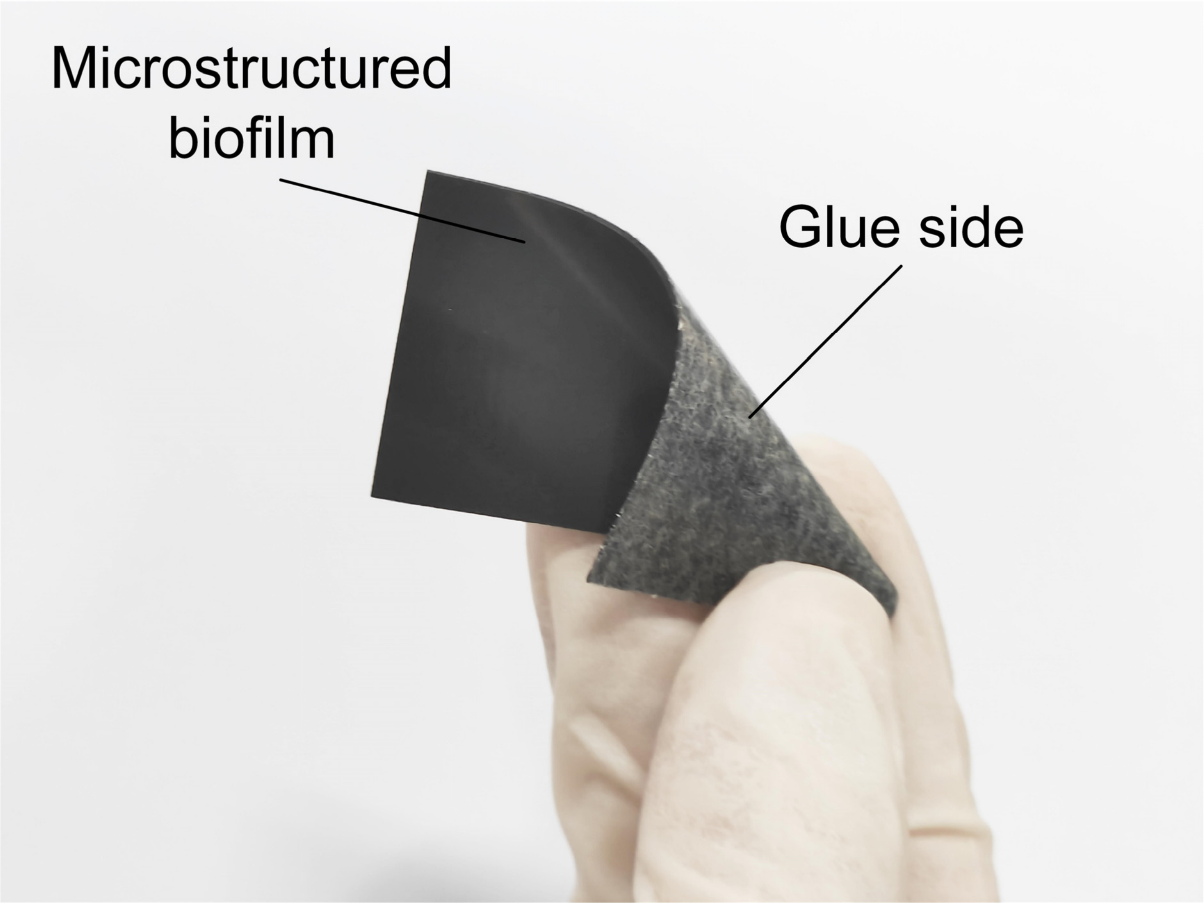

Fig. S3. Microstructured biofilm with glue on the back.

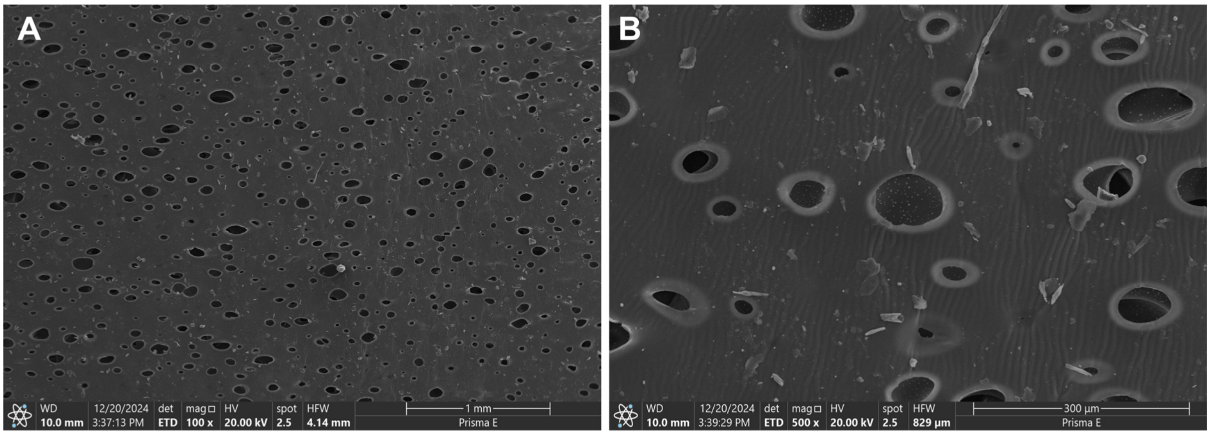

Fig. S4. Scanning electron microscope (SEM) characterization of microstructure biofilm morphology. (A) SEM image with a horizontal field (HFW) width of 4.14 mm. (B) SEM image with an HFW of 829 μm.

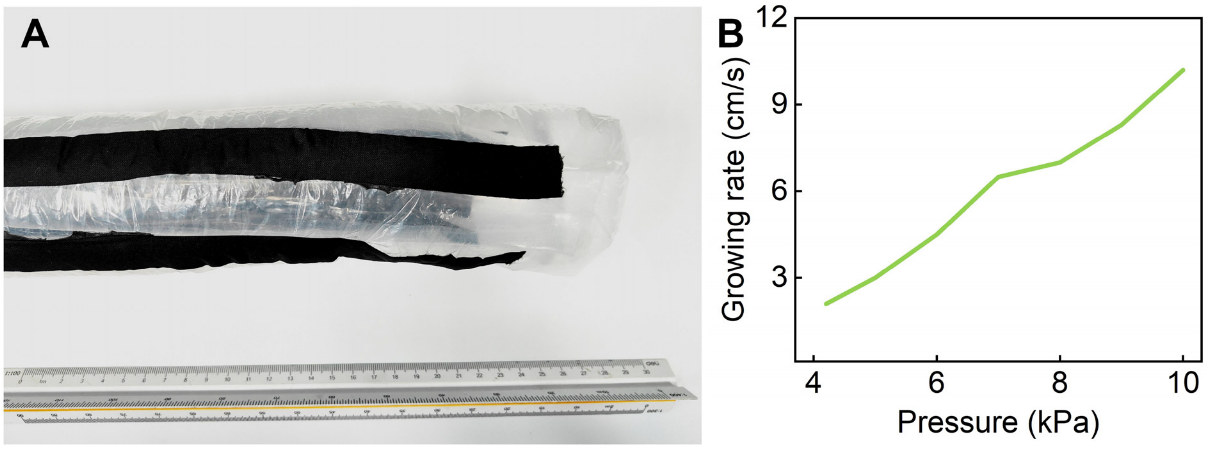

Fig. S5. Growth rate test of the soft climbing robot. (A) Actual test image. (B) Growth rate under different pressures.

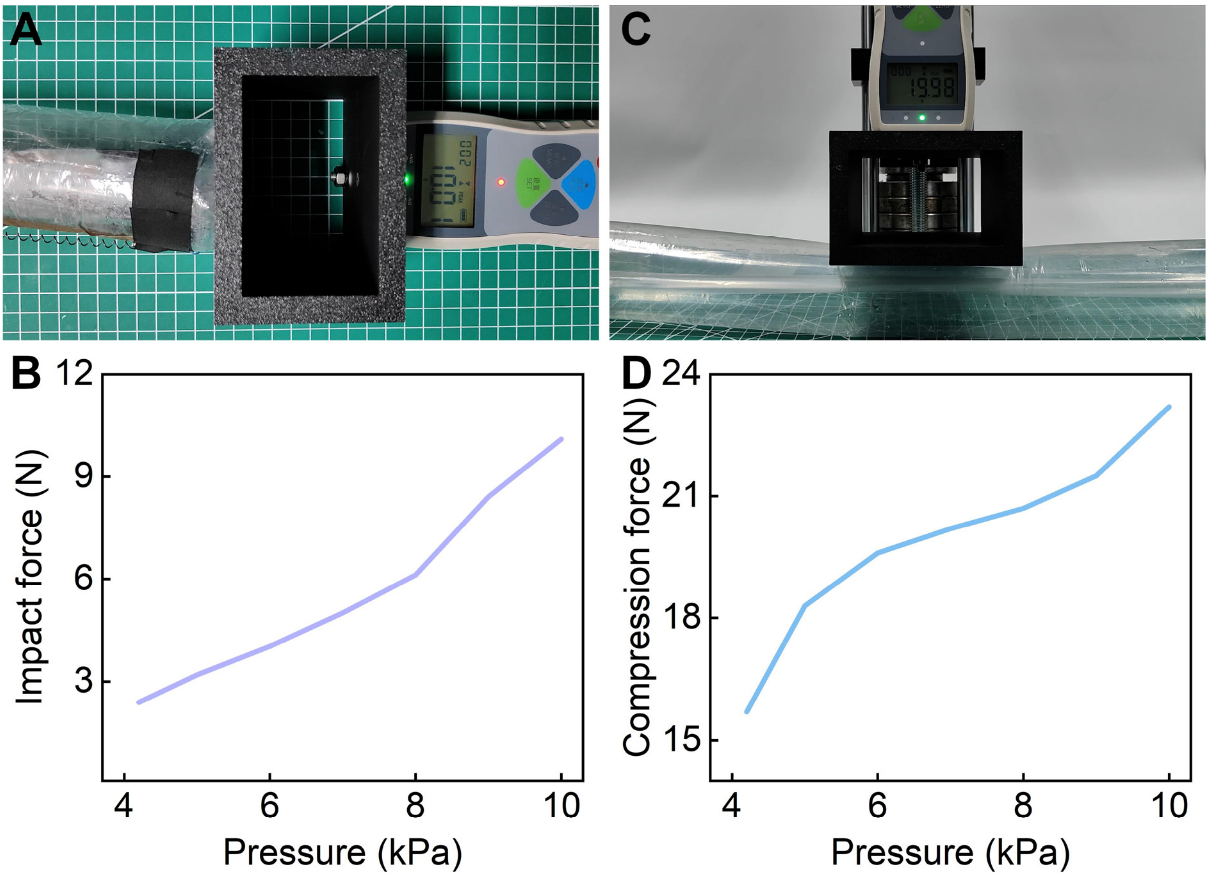

Fig. S6. Impact and compression forces at different pressures. (A and B) Impact and force at different pressures. (C and D) Compression forces at different pressures.

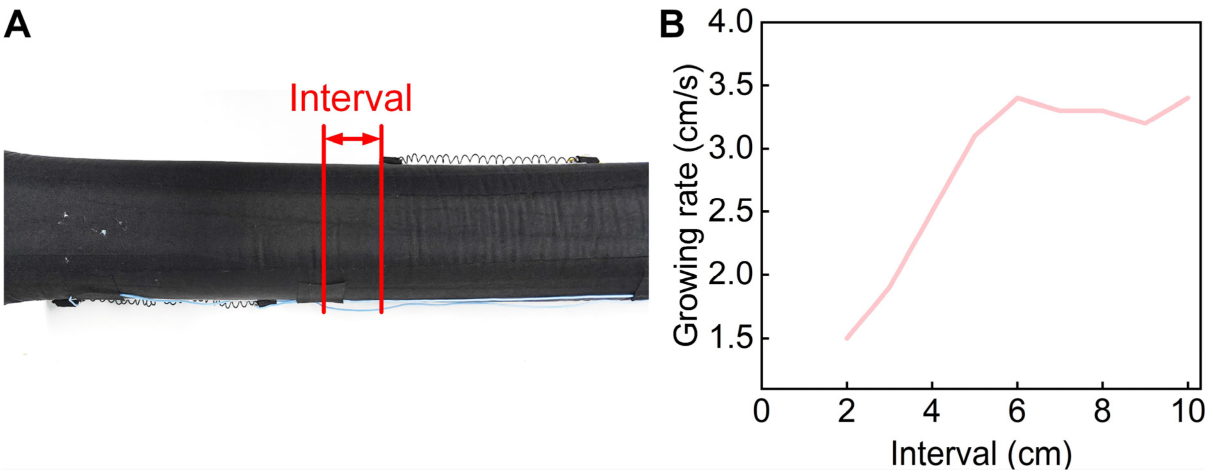

Fig. S7. The test of SMA spring intervals. (A) The interval of the SMA spring. (B) Growth rate under different SMA spring intervals.

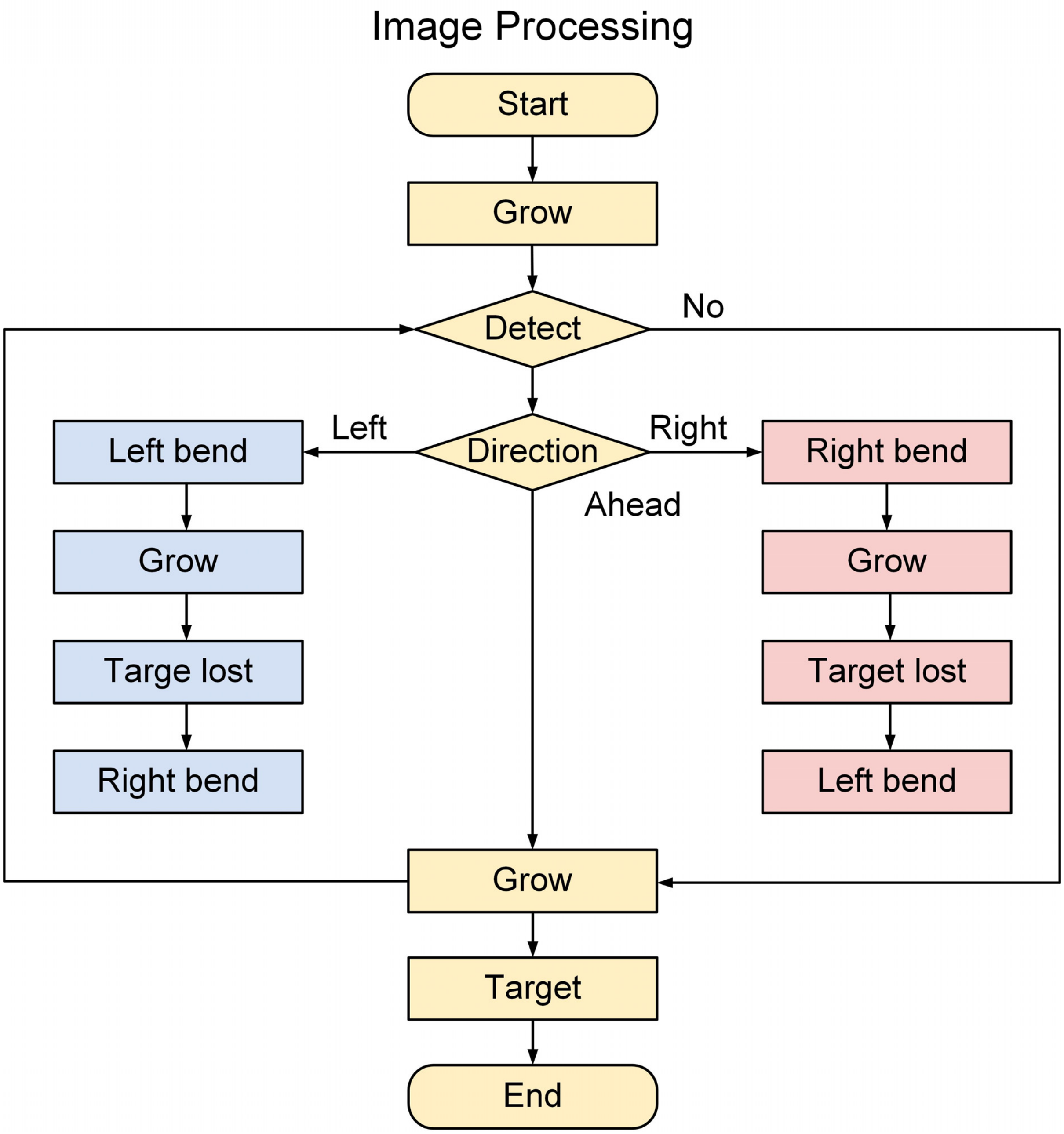

Fig. S8. Movement flow chart of the soft climbing robot.

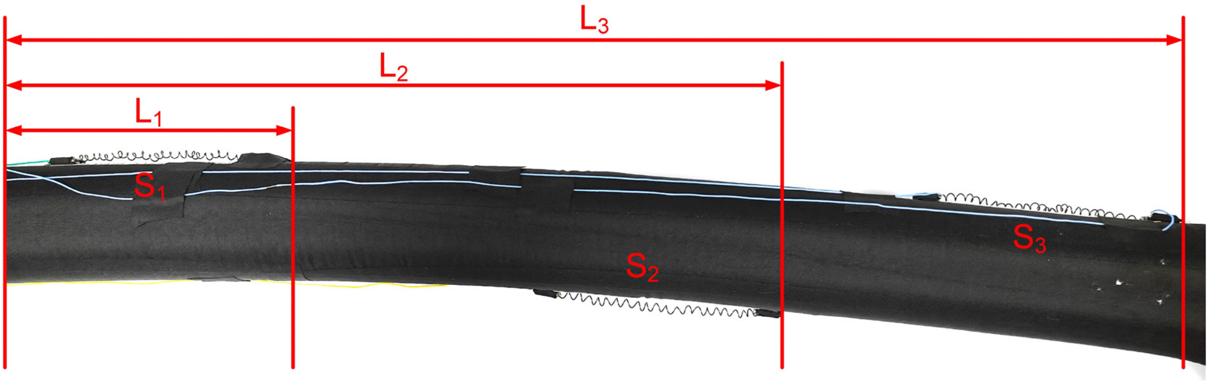

Fig. S9. Segmentation rules for SMA springs.

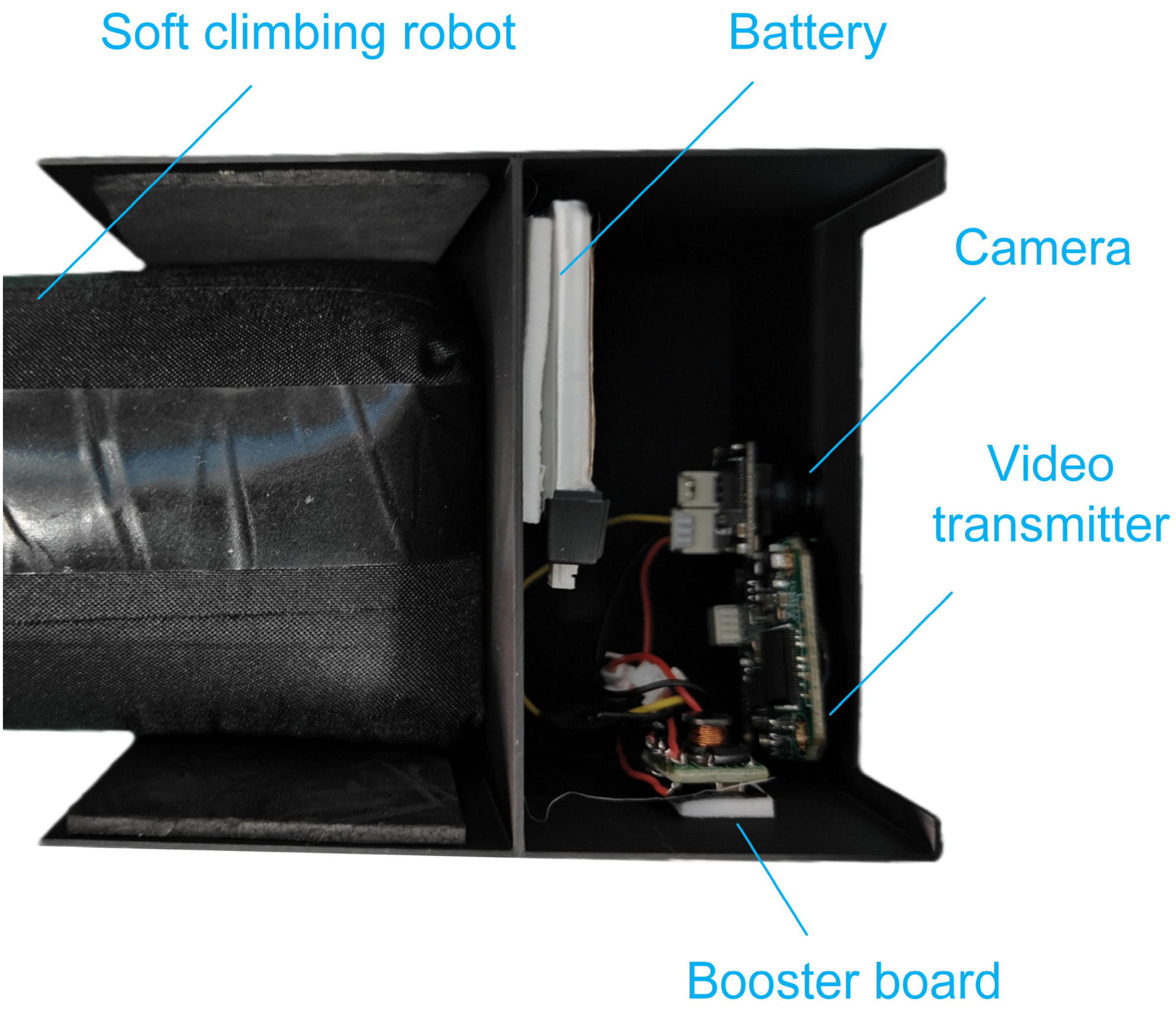

Fig. S10. Camera module structure and installation.

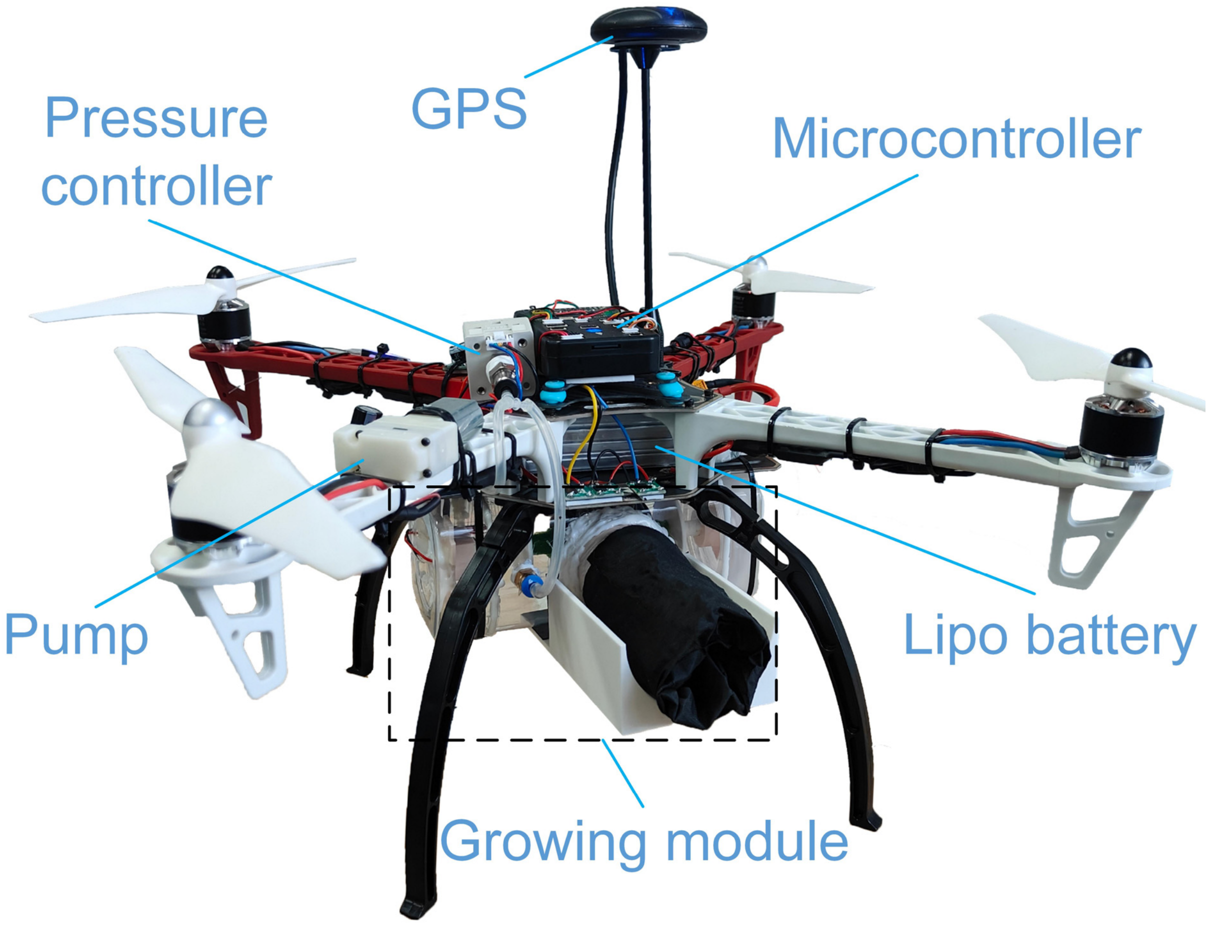

Fig. S11. Picture of the combination of a drone and a soft climbing robot.

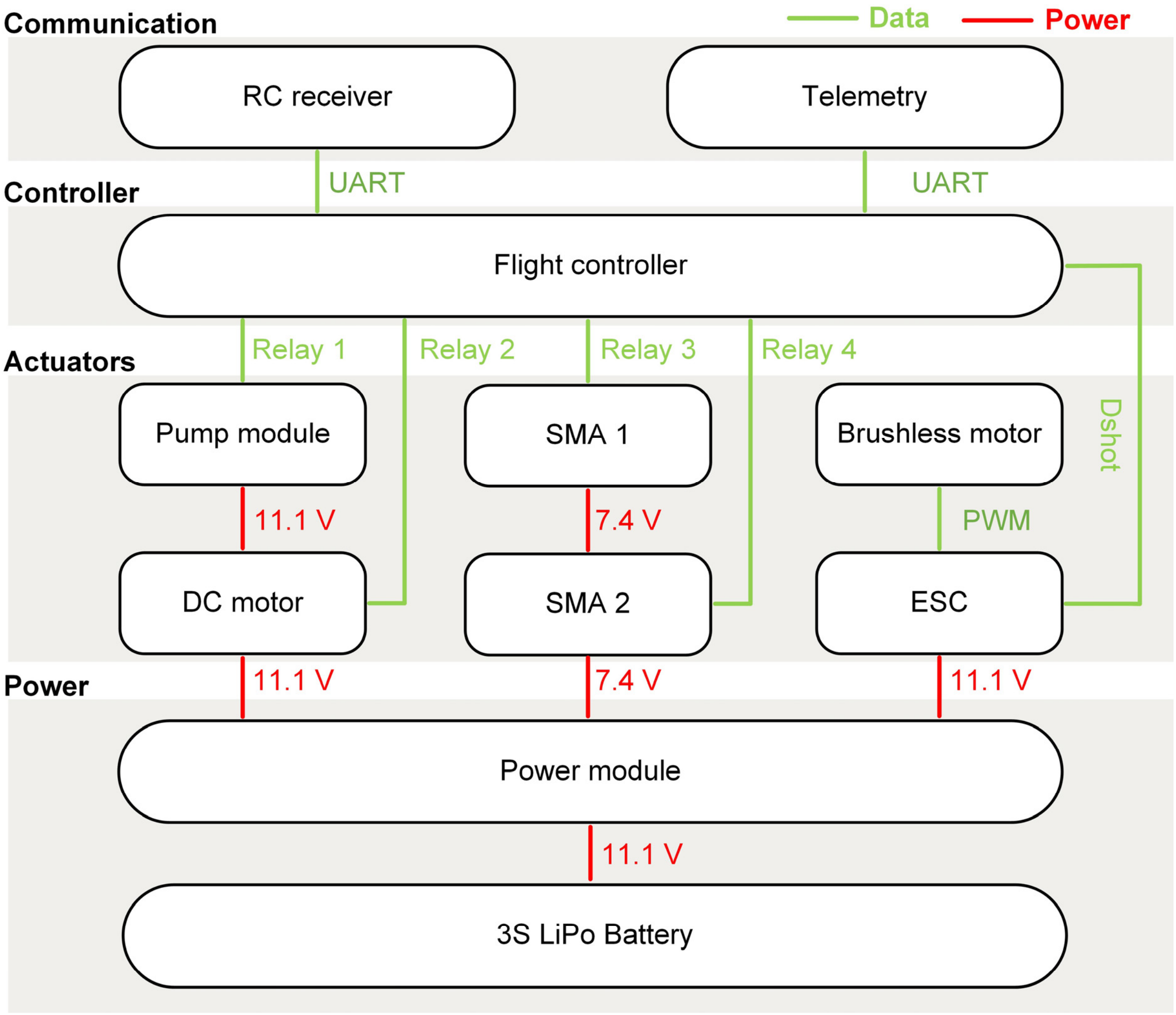

Fig. S12. Circuit connection between the drone and the soft climbing robot.

Table S1. Weight of the soft climbing robot on the drone.

	Mass (g)
Growing module	164.8
Valve	15
Pump	31.7
Pressure controller	45.8
Camera module	14.6
Others	12.1
Total	284

Movie S1. Soft climbing robot climbing on surfaces of different materials.

Movie S2. Soft climbing robot climbing on surfaces with complex structures.

Movie S3. Growing and bending of the soft climbing robot.

Movie S4. 3D bending of the soft climbing robot.

Movie S5. Contraction of the soft climbing robot.

Movie S6. Impact and compression testing of the soft climbing robot.

Movie S7. Soft climbing robot for visual inspection.

Movie S8. Aerial growth of the soft climbing robot.

Movie S9. Soft climbing robot combined with a drone.

REFERENCES AND NOTES

1. T. Yue, H. Bloomfield-Gadêlha, J. Rossiter, Snail-inspired water-enhanced soft sliding suction for climbing robots. *Nat. Commun.* **15**, 4038 (2024).

2. W. Pang, S. Xu, J. Wu, R. Bo, T. Jin, Y. Xiao, Z. Liu, F. Zhang, X. Cheng, K. Bai, H. Song, Z. Xue, L. Wen, Y. Zhang, A soft microrobot with highly deformable 3D actuators for climbing and transitioning complex surfaces. *Proc. Natl. Acad. Sci. U.S.A.* **119**, e2215028119 (2022).

3. Y. Wu, X. Dong, J.-K. Kim, C. Wang, M. Sitti, Wireless soft millirobots for climbing three-dimensional surfaces in confined spaces. *Sci. Adv.* **8**, eab3431 (2022).

4. C. Tang, B. Du, S. Jiang, Q. Shao, X. Dong, X.-J. Liu, H. Zhao, A pipeline inspection robot for navigating tubular environments in the sub-centimeter scale. *Sci. Robot.* **7**, eabm8597 (2022).

5. B. Tao, Z. Gong, H. Ding, Climbing robots for manufacturing. *Natl. Sci. Rev.* **10**, nwad042 (2023).

6. S. Hong, Y. Um, J. Park, H.-W. Park, Agile and versatile climbing on ferromagnetic surfaces with a quadrupedal robot. *Sci. Robot.* **7**, eadd1017 (2022).

7. M. Zheng, D. Wang, D. Zhu, S. Cao, X. Wang, M. Zhang, PiezoClimber: Versatile and self-transitional climbing soft robot with bioinspired highly directional footpads. *Adv. Funct. Mater.* **34**, 2308384 (2024).

8. W. Hu, G. Z. Lum, M. Mastrangeli, M. Sitti, Small-scale soft-bodied robot with multimodal locomotion. *Nature* **554**, 81–85 (2018).

9. A. Parness, M. Frost, N. Thatte, J. P. King, K. Witkoe, M. Nevarez, M. Garrett, H. Aghazarian, B. Kennedy, Gravity-independent rock-climbing robot and a sample acquisition tool with microspine grippers. *J. Field Robot.* **30**, 897–915 (2013).

10. Q. Hu, E. Dong, D. Sun, Soft modular climbing robots. *IEEE Trans. Robot.* **39**, 399–416 (2023).

11. M. Xuefeng, Y. Liu, L. Junkao, D. Jie, Crabbot: A pole-climbing robot driven by piezoelectric stack. *IEEE Trans. Robot.* **38**, 765–778 (2022).

12. Z. Li, Z. Li, L. M. Tam, Q. Xu, Design and development of a versatile quadruped climbing robot with obstacle-overcoming and manipulation capabilities. *IEEE/ASME Trans. Mechatron.* **28**, 1649–1661 (2023).

13. D. Xie, J. Liu, R. Kang, S. Zuo, Fully 3D-printed modular pipe-climbing robot. *IEEE Robot. Autom. Lett.* **6**, 462–469 (2021).

14. J. Hu, X. Han, Y. Tao, S. Feng, A magnetic crawler wall-climbing robot with capacity of high payload on the convex surface. *Robot. Auton. Syst.* **148**, 103907 (2022).

15. T. Yue, W. Si, A. Keller, C. Yang, H. Bloomfield-Gadêlha, J. Rossiter, Bioinspired multiscale adaptive suction on complex dry surfaces enhanced by regulated water secretion. *Proc. Natl. Acad. Sci. U.S.A.* **121**, e2314359121 (2024).

16. G. Lee, G. Wu, S. H. Kim, J. Kim, T. Seo, Combot: Compliant climbing robotic platform with transitioning capability and payload capacity in *2012 IEEE International Conference on Robotics and Automation (ICRA)* (IEEE, 2012), pp. 2737–2742.

17. E. W. Hawkes, D. L. Christensen, M. R. Cutkosky, Vertical dry adhesive climbing with a 100× bodyweight payload” in *2015 IEEE International Conference on Robotics and Automation (ICRA)* (IEEE, 2015), pp. 3762–3769.

18. G. Gu, J. Zou, R. Zhao, X. Zhao, X. Zhu, Soft wall-climbing robots. *Sci. Robot.* **3**, eaat2874 (2018).

19. C. Darwin, The movements and habits of climbing plants. *Nature* **13**, 65–66 (1875).

20. Y. Wu, X. Zhao, M. Zhang, Adhesion mechanics of ivy nanoparticles. *J. Colloid Interface Sci.* **344**, 533–540 (2010).

21. K. Jonsson, Y. Ma, A.-L. Routier-Kierzkowska, R. P. Bhalerao, Multiple mechanisms behind plant bending. *Nat. Plants* **9**, 13–21 (2023).

22. E. Del Dottore, A. Mondini, N. Rowe, B. Mazzolai, A growing soft robot with climbing plant–inspired adaptive behaviors for navigation in unstructured environments. *Sci. Robot.* **9**, eadi5908 (2024).

23. N. D. Naclerio, A. Karsai, M. Murray-Cooper, Y. Ozkan-Aydin, E. Aydin, D. I. Goldman, E. W. Hawkes, Controlling subterranean forces enables a fast, steerable, burrowing soft robot. *Sci. Robot.* **6**, eabe2922 (2021).

24. M. M. Hausladen, B. Zhao, M. S. Kubala, L. F. Francis, T. M. Kowalewski, C. J. Ellison, Synthetic growth by self-lubricated photopolymerization and extrusion inspired by plants and fungi. *Proc. Natl. Acad. Sci. U.S.A.* **119**, e2201776119 (2022).

25. P. Berthet-Rayne, S. M. H. Sadati, G. Petrou, N. Patel, S. Giannarou, D. R. Leff, C. Bergeles, MAMMOBOT: A miniature steerable soft growing robot for early breast cancer detection. *IEEE Robot. Autom. Lett.* **6**, 5056–5063 (2021).

26. A. Ataka, T. Abrar, F. Putzu, H. Godaba, K. Althoefer, Observer-based control of inflatable robot with variable stiffness in *2020 IEEE/RSJ International Conference on Intelligent Robots and Systems (IROS)* (IEEE/RSJ, 2020), pp. 8646–8652.

27. J. D. Greer, T. K. Morimoto, A. M. Okamura, E. W. Hawkes, Series pneumatic artificial muscles (sPAMs) and application to a soft continuum robot, in *2017 IEEE International Conference on Robotics and Automation (ICRA)* (IEEE, 2017), pp. 5503–5510.

28. J. D. Greer, T. K. Morimoto, A. M. Okamura, E. W. Hawkes, A soft, steerable continuum robot that grows via tip extension. *Soft Robot.* **6**, 95 108 (2019).

29. N. D. Naclerio, E. W. Hawkes, Simple, low-hysteresis, foldable, fabric pneumatic artificial muscle. *IEEE Robot. Autom. Lett.* **5**, 3406–3413 (2020).

30. A. M. Kübler, S. U. Rivera, F. B. Raphael, J. Förster, R. Siegwart, A. M. Okamura, "A multi-segment, soft growing robot with selective steering" in *2023 IEEE International Conference on Soft Robotics (RoboSoft)* (IEEE, 2023), pp. 1–7.

31. T. Takahashi, M. Watanabe, K. Abe, K. Tadakuma, N. Saiki, M. Konyo, S. Tadokoro, Inflated bendable eversion cantilever mechanism with inner skeleton for increased stiffness. *IEEE Robot. Autom. Lett.* **8**, 168–175 (2023).

32. D.-G. Lee, N. G. Kim, J.-H. Ryu, High-curvature consecutive tip steering of a soft growing robot for improved target reachability in *2023 IEEE/RSJ International Conference on Intelligent Robots and Systems (IROS)* (IEEE, 2023), pp. 6477–6483.

33. D. A. Haggerty, N. D. Naclerio, E. W. Hawkes, Hybrid vine robot with internal steering-reeling mechanism enhances system-level capabilities. *IEEE Robot. Autom. Lett.* **6**, 5437–5444 (2021).

34. Y. Satake, A. Takanishi, H. Ishii, Novel growing robot with inflatable structure and heat-welding rotation mechanism. *IEEE/ASME Trans. Mechatron.* **25**, 1869–1877 (2020).

35. M. M. Coad, R. P. Thomasson, L. H. Blumenschein, N. S. Usevitch, E. W. Hawkes, A. M. Okamura, Retraction of soft growing robots without buckling. *IEEE Robot. Autom. Lett.* **5**, 2115–2122 (2020).

36. N. G. Kim, D. Seo, S. Park, J.-H. Ryu, Self-retractable soft growing robots for reliable and fast retraction while preserving their inherent advantages. *IEEE Robot. Autom. Lett.* **9**, 1082–1089 (2024).

37. P. Li, Y. Zhang, G. Zhang, D. Zhou, L. Li, A bioinspired soft robot combining the growth adaptability of vine plants with a coordinated control system. *Research* **2021**, 9843859 (2021).

38. Y. Tian, N. Pesika, H. Zeng, K. Rosenberg, B. Zhao, P. McGuiggan, K. Autumn, J. Israelachvili, Adhesion and friction in gecko toe attachment and detachment. *Proc. Natl. Acad. Sci. U.S.A.* **103**, 19320–19325 (2006).

39. K. Qin, W. Tang, Y. Zhong, Y. Liu, H. Xu, P. Zhu, D. Yan, H. Yang, J. Zou, An aerial–aquatic robot with tunable tilting motors capable of multimode motion. *Adv. Intell. Syst.* **5**, 2300193 (2023).

40. X. Guo, W. Tang, K. Qin, Y. Zhong, H. Xu, Y. Qu, Z. Li, Q. Sheng, Y. Gao, H. Yang, J. Zou, Powerful UAV manipulation via bioinspired self-adaptive soft self-contained gripper. *Sci. Adv.* **10**, eadn6642 (2024).

41. S. D. de Rivaz, B. Goldberg, N. Doshi, K. Jayaram, J. Zhou, R. J. Wood, Inverted and vertical climbing of a quadrupedal microrobot using electroadhesion. *Sci. Robot.* **3**, eaau3038 (2018).

42. L. Yao, X. Peng, H. Zhu, Q. Zhang, S. Zhu, Robust transient semi-glue tape: Ultrastrong adhesion empowered by water activation and self-locking. *Adv. Mater.* **36**, 2405511 (2024).
